# Pirin, an Nrf2-Regulated Protein, Is Overexpressed in Human Colorectal Tumors

**DOI:** 10.3390/antiox11020262

**Published:** 2022-01-28

**Authors:** Ying Zhang, Elena V. Knatko, Maureen Higgins, Sharadha Dayalan Naidu, Gillian Smith, Tadashi Honda, Laureano de la Vega, Albena T. Dinkova-Kostova

**Affiliations:** 1 Jacqui Wood Cancer Centre, Division of Cellular Medicine, School of Medicine, University of Dundee, Dundee DD1 9SY, UK; Y.F.Zhang@dundee.ac.uk (Y.Z.); e.knatko@dundee.ac.uk (E.V.K.); m.higgins@dundee.ac.uk (M.H.); s.z.dayalannaidu@dundee.ac.uk (S.D.N.); g.smith@dundee.ac.uk (G.S.); l.delavega@dundee.ac.uk (L.d.l.V.); 2 Department of Chemistry, Institute of Chemical Biology & Drug Discovery, Stony Brook University, Stony Brook, New York, NY 11794, USA; tadashi.honda@stonybrook.edu; 3 Department of Pharmacology and Molecular Sciences, Johns Hopkins University School of Medicine, Baltimore, MD 21205, USA; 4 Department of Medicine, Johns Hopkins University School of Medicine, Baltimore, MD 21205, USA

**Keywords:** AKR1B10, AKR1C1, colorectal cancer, DLD1, Nrf2, NQO1, pirin

## Abstract

The evolutionary conserved non-heme Fe-containing protein pirin has been implicated as an important factor in cell proliferation, migration, invasion, and tumour progression of melanoma, breast, lung, cervical, prostate, and oral cancers. Here we found that pirin is overexpressed in human colorectal cancer in comparison with matched normal tissue. The overexpression of pirin correlates with activation of transcription factor nuclear factor erythroid 2 p45-related factor 2 (Nrf2) and increased expression of the classical Nrf2 target NAD(P)H:quinone oxidoreductase 1 (NQO1), but interestingly and unexpectedly, not with expression of the aldo-keto reductase (AKR) family members AKR1B10 and AKR1C1, which are considered to be the most overexpressed genes in response to Nrf2 activation in humans. Using pharmacologic and genetic approaches to either downregulate or upregulate Nrf2, we show that pirin is regulated by Nrf2 in human and mouse cells and in the mouse colon in vivo. The small molecule pirin inhibitor TPhA decreased the viability of human colorectal cancer (DLD1) cells, but this decrease was independent of the levels of pirin. Our study demonstrates the Nrf2-dependent regulation of pirin and encourages the pursuit for specific pirin inhibitors.

## 1. Introduction

Pirin is a highly evolutionary conserved non-heme Fe-containing family member of the cupin superfamily of proteins, which in its oxidized (Fe^+++^-bound) form, binds to the RelA (p65) subunit and enhances the activity of transcription factor NFκB [1]. Pirin can also form complexes with B-cell lymphoma 3-encoded protein (Bcl3) and NFκB1 (p50) on NFκB DNA binding sites [2]. Furthermore, several reports have implicated pirin as an important factor in cancer cell proliferation, migration, and tumour progression in the context of melanoma, breast, lung, cervical, prostate, and oral cancer [3,4,5,6,7].

Although the molecular details of how pirin is regulated are incompletely understood, increasing evidence points to the role of transcription factor nuclear factor erythroid 2 p45-related factor 2 (Nrf2, gene name *NFE2L2*) in mediating the gene expression of pirin. Thus, a gene expression study comparing the small airway epithelium of healthy non-smokers to that of healthy smokers using samples obtained by fiberoptic bronchoscopy identified pirin as a “smoking-responsive Nrf2-modulated gene”, and further showed that the promoter region of the human PIR gene contains two antioxidant response elements (AREs), the DNA sequences to which Nrf2 binds, located at −3209 bp and −5566 bp upstream of the transcription start site [8]. It was also reported that the basal expression of pirin in HeLa cells is regulated by Nrf2 [9]. A chemical proteomics approach suggested pirin as one of the proteins regulated by Nrf2 in non-small cell lung cancer cell lines [10]. Recent studies have implicated Nrf2 in the overexpression of pirin caused by the high-risk human papillomavirus (HR-HPV) E7 oncoprotein [6], as well as by small-molecule inducers of ferroptosis [11]. Interestingly, RNA-sequencing (RNA-seq) expression profiling of HEK293T cells has shown that pirin expression is induced upon treatment with the electrophile 4-hydroxynonenal (HNE), but not by the oxidant H_2_O_2_ [12]. Both HNE and H_2_O_2_ induce Nrf2-mediated transcription following chemical modification of specific cysteine sensors of Kelch-like ECH associated protein 1 (Keap1), the principal negative regulator of Nrf2 [13,14]. Curiously, inhibition of the expression of pirin was seen when cervical carcinoma cells were exposed to curcumin [15], a dietary plant Michael reaction acceptor, which we and others have found to activate Nrf2 [16,17,18].

Using proteomics, we identified pirin as one of the differentially abundant proteins in intestinal organoid preparations from wild-type, Keap1-knockdown and Nrf2-knockout mice [19]. In this study, we employed pharmacologic Nrf2 activators, as well as genetic approaches to either downregulate or upregulate Nrf2, which demonstrated that pirin is regulated by Nrf2 in human and mouse cells, and in the mouse colon in vivo. Furthermore, we found that pirin is overexpressed in human colorectal tumours, and this overexpression correlates with Nrf2 activation.

## 2. Materials and Methods

### 2.1. Materials

The chemicals and reagents were obtained from common commercial suppliers. The synthesis of (±)-TBE-31 was as described [20]. Sulforaphane was purchased from LKT Labs. Triphenyl compound A (TPhA) was purchased from Sigma, Dorset, UK.

### 2.2. Cells

The isogenic human colorectal cancer cell lines DLD1 (WT Nrf2, Nrf2-knockout and Nrf2-gain-of-function), human lung cancer cell lines A549 (hyper-Nrf2 and Nrf2-knockout), human osteosarcoma cell lines U2OS (WT Nrf2 and Nrf2-knockout) were generated as described previously [21]. These cell lines were grown in Dulbecco’s Modified Eagle Medium (DMEM, Gibco) that contains L-glutamine, sodium pyruvate, and high D-glucose content (4.5 g/L) supplemented with 10% (*v*/*v*) heat-inactivated fetal bovine serum (FBS, Gibco). The K-Ras^WT/−^ and K-Ras^G13D/−^ DLD1 cell lines, which had been generated from the parental K-Ras^WT/G13D^ DLD1 cell line following deletion of the mutant K-Ras^G13D^ allele [22,23], were a kind gift from Bert Vogelstein (Johns Hopkins University School of Medicine, Baltimore, MD, USA) and grown in RPMI 1640 Medium (Gibco) supplemented with 10% FBS. The normal human lung fibroblast cell line IMR90 was from the American Type Culture Collection (ATCC) and grown in DMEM supplemented with 20% (*v*/*v*) heat-inactivated FBS. The human colorectal cancer cell line Caco2, a kind gift from Colin Henderson (University of Dundee School of Medicine, Dundee, UK), were cultured in DMEM supplemented with 10% (*v*/*v*) FBS and 1% (*v*/*v*) MEM Non-essential Amino Acid Solution (Sigma). Primary cultures of mouse embryonic fibroblast (MEF) cells were derived from wild-type, Nrf2-knockout, and Keap1-knockdown Skh-1 hairless mice [24]. The plastic dishes were coated for 30 min with 0.1% (*w*/*v*) gelatin before use and the MEF cells were grown in Iscove’s modified Dulbecco’s medium (with L-glutamine) (IMDM) supplemented with human recombinant EGF (10 ng/mL), 1 × insulin/transferring/selenium, and 10% (*v*/*v*) heat-inactivated FBS. All cell cultures were kept in an incubator at 37 °C, 5% CO_2_. Routine testing ensured that they were mycoplasma-free.

For treatments of cells, TBE-31 and sulforaphane were dissolved in acetonitrile, whereas TPhA was dissolved in DMSO. The respective stock solutions were then diluted in the cell culture medium at a ratio of 1:1000 to achieve the appropriate final concentrations indicated in the text. The final concentration of acetonitrile or DMSO in the cell culture medium was 0.1% (*v*/*v*).

### 2.3. Animals

The regulations described in the UK Animals (Scientific Procedures) Act 1986 and the institutional guidelines were strictly observed for all mouse experiments. Mice were bred and maintained with free access to water and food (pelleted RM1 diet from SDS Ltd., Witham, Essex, UK), on a 12-h light/12-h dark cycle, 35% humidity at the Medical School Resource Unit of the University of Dundee. For preparation of intestinal organoids and colon pirin expression analysis, wild-type (WT), Nrf2-knockout (Nrf2^−/−^, Nrf2-KO) and Keap1-knockdown (Keap1^flox/flox^, Keap1-KD) C57BL/6 mice were used.

### 2.4. Organoids

Organoids were generated from isolated intestinal crypts and cultured with Matrigel as described [25,26]. The culture medium was Advanced DMEM/F12 (ADF, Gibco) medium supplemented with 10 mM HEPES, 20 mM Glutamax, 1 mM N-acetylcysteine, N2 (Gemini), B27 (Gibco), penicillin-streptomycin (Gibco), EGF (50 ng/mL, Gibco), Noggin (100 ng/mL, eBioscience, Altrincham, UK), and R-Spondin conditioned medium (1:4) at 37 °C, 5% CO_2_. For passaging, the organoids were washed with cold ADF medium, mechanically broken using a pipette, washed again, mixed with fresh Matrigel, and reseeded on 24-well plates. For gene expression analysis, RNA was extracted from organoids growing in 3 individual wells.

### 2.5. TBE-31 Oral Gavage

For oral gavage administration to mice, a stock solution of TBE-31 in DMSO was prepared and diluted (1:140) in corn oil to achieve a dose of 5 nmol/g body weight. Wild-type (WT) and Nrf2-knockout (Nrf2^−/−^, Nrf2-KO) C57BL/6 female mice were recruited to the study when they were 14 weeks old.

Mice were randomly allocated to each treatment group (*n* = 4–5) and administered with TBE-31 or vehicle (0.7% DMSO in corn oil), at 24 h intervals, for 3 days. Animals were sacrificed at 24 h after the last treatment (mice were fasted for 4 h before harvesting). Colon tissues were collected and snap frozen in liquid nitrogen for further analysis.

### 2.6. Patient Samples

Human tissues were from patients with a histologically confirmed diagnosis of colorectal cancer who were recruited to a prior study (Weidlich et al., 2011). Following approval by the Tayside Tissue Bank Research Ethics Committee, a sub-committee of the Tayside Committee on Medical Research Ethics, in this study, we analyzed tumours (*n* = 48) from both female and male patients, for which matched normal tissue was also available. Patient demographics and tumour pathology are summarized in Table 1.

### 2.7. Real-Time Quantitative PCR

For tissues from human patients, frozen tissue samples were disrupted in a Tissue Lyser II (Qiagen, Manchester, UK) and RNA was extracted using the RNeasy Mini Plus Kit (Qiagen) following manufacturer’s instructions. The isolated RNA was quantified using the Qubit RNA BR assay (Thermo Fisher, Altrincham, UK) and analysed on a TapeStation 2200 using the RNA ScreenTape assay (Agilent). Total RNA from cells and mouse tissues was extracted using RNeasy Kit (Qiagen) and quantified using NanoDrop™ Spectrophotometer (Thermo Fisher). Total RNA (500 ng) was reverse-transcribed into cDNA using Omniscript RT Kit (Qiagen). Real-time PCR was carried out on Applied Biosystems QuantStudio™ 5 Real-Time PCR System. The TaqManTM Gene Expression Assay IDs (Thermo Fisher) used are listed in Table 2 below.

### 2.8. Transfection with Small Interfering RNA (siRNA)

DLD1 cells were transfected with 20 nM ON-TARGET plus Smart Pool siRNA against human PIR (L-012613-00-0005, Horizon Discovery, Waterbeach, UK) or ON-TARGET plus Non-targeting Control Pool (D-001810-10-50, Horizon Discovery) using Lipofectamine^®^ RNAiMAX (Thermo Scientific, Waltham, MA, USA). In general, siRNA targeting PIR/non-targeting control and Lipofectamine^®^ RNAiMAX were mixed in Opti-MEM (Gibco, Thermo Scientific) and incubated for 20 min at room temperature. In parallel, DLD1 cells were trypsinized and diluted to a cell density of 6 × 10^4^ cells per ml of medium, and RNAiMAX/siRNA/Opti-MEM (200 µL per ml) was added. The cell suspension was mixed gently and the cells were seeded into 96-well plates for Alamar Blue cell viability assay or into 6-well plates for real-time quantitative PCR and immunoblotting analysis.

### 2.9. Alamar Blue Cell Viability Assay

DLD1 cells (5 × 10^3^ per well) seeded in white flat-bottomed 96-well plates (Nunc) were treated as indicated in the text. Four hours before the end of each treatment, 10 µL of Alamar Blue dye solution (AbdSerotec, Oxford, UK) was added to the cell culture media (100 µL) within each well, and the plates were returned to the incubator. After 4 h, the fluorescence of the reduced probe was measured (ex. 560 nm/em. 590 nm, SpectraMax M2, Molecular Devices, San Jose, CA, USA), and the cell viability was calculated. Ten replicate wells were analysed for each condition.

### 2.10. Cell Migration Assay

Pirin siRNA- and siRNA negative control-transfected DLD1 cells (1.5 × 10^4^ cells per well, 6 replicate wells for each condition) were seeded in 96-well ImageLock plates (Essen BioScience, Royston, UK) and grown for 48 h to confluence. After this, the cells were starved in serum-free medium overnight. The cell monolayers were then scratched using a 96-pin WoundMaker according to the manufacturer’s instructions (Essen BioScience). The wells were washed with PBS to remove any debris and incubated in DMEM with 0.2% FBS. Wound images were automatically acquired and registered by the IncuCyte software system (Essen BioScience). Typical kinetic updates were recorded at 2-h intervals for the duration of the experiment (48 h). The data were processed and analyzed by the IncuCyte™ software and are presented as the Relative Wound Density.

### 2.11. Immunoblotting

Cells were lysed in 150 µL of non-reducing sample buffer (50 mM Tris-HCl pH 6.8, 2% (*w*/*v*) sodium dodecyl sulfate (SDS), 10% (*v*/*v*) glycerol, and 0.02% (*w*/*v*) Bromophenol blue). Whole-cell lysates were then collected in Eppendorf tubes, boiled at 100 °C for 5 min, and sonicated using Vibra-Cell ultrasonic processor (Sonic, Sevenoaks, UK) for 20 s at 20% amplitude. The BCA assay (Thermo) was used to determine protein concentrations. Next, 2-Mercaptoethanol (Sigma) was added to a final concentration of 6% (*v*/*v*). Proteins were resolved by SDS/PAGE, and transferred to 0.45 µm nitrocellulose (NC) membranes (Cytiva, Marlborough, MA, USA). Solutions of primary antibodies were prepared in 5% milk in PBST. The following antibodies and dilutions were used: rat monoclonal anti-pirin (1E8), 1:1000, CST; and mouse monoclonal anti-β-actin, 1: 1:20,000, Sigma. Blocked NC membranes were incubated with the primary antibody solutions in 50-mL tubes at 4 °C overnight, with continuous rotation. After this, the NC membranes were washed and incubated with IRDye fluorescent dyes conjugated goat anti-mouse-680RD or goat anti-rat-800 CW, 1:15,000 (LI-COR). All immunoblots shown are representative of 2–3 independent experiments.

### 2.12. Statistical Analysis

Statistical analysis was performed using GraphPad Prism 8 software (GraphPad Software, Inc., La Jolla, CA, USA). The differences between groups were determined by Student’s *t*-test. The data are presented as the mean ± SD, and the significance level was set at *p* < 0.05. For correlation analysis, non-parametric test Spearman and Kendall rank-based correlation were used. The correlation between two variables was considered to be significant when the *p*-value is <0.05.

## 3. Results

### 3.1. Pirin Is a Transcriptional Target of Nrf2 in Human Cell Lines

**Figure 1:**
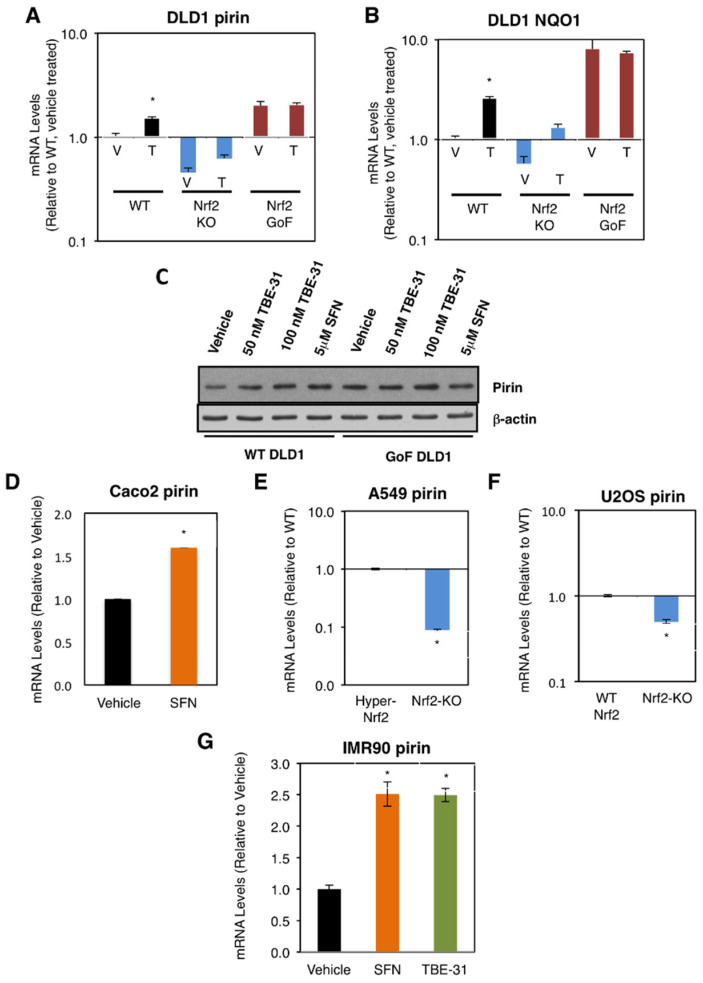
Pirin is a transcriptional target of Nrf2 in human cells. (**A**,**B**) mRNA levels for pirin (**A**) and NQO1 (**B**) in Nrf2-wild-type (WT), Nrf2-knockout (Nrf2-KO), and Nrf2 gain-of-function mutant (Nrf2-GoF) human DLD1 colorectal cancer cells treated with vehicle (V) or 10 nM TBE-31 (T) for 16 h. (**C**) Protein levels for pirin in Nrf2-wild-type (WT) and Nrf2 gain-of-function mutant (Nrf2-GoF) human DLD1 colorectal cancer cells treated with vehicle, 50 nM TBE-31, 100 nM TBE-31, or 5 μM sulforaphane (SFN) for 16 h. (**D**) mRNA levels for pirin in Caco2 colorectal cancer cells treated with vehicle or 5 μM SFN for 24 h. (**E**) mRNA levels for pirin in A549 cells (high Nrf2 levels, “Hyper-Nrf2”) and their Nrf2-knockout (Nrf2-KO) mutant counterparts. (**F**) mRNA levels for pirin in U2OS cells (normal Nrf2 levels, WT Nrf2) and their Nrf2-knockout (Nrf2-KO) mutant counterparts. (**G**) mRNA levels for pirin in IMR90 normal human lung fibroblasts treated with vehicle, 5 μM SFN or 100 nM TBE-31 for 16 h. * *p* < 0.05. The TaqMan data were normalized using human hypoxanthine phosphoribosyltransferase 1 (Hprt1) as an internal control.

We first compared the mRNA levels for pirin in the human colorectal cancer cell line DLD1 (Nrf2-WT) as well as Nrf2-knockout (Nrf2-KO) and Nrf2-gain-of-function (Nrf2-GoF) mutant isogenic lines that had been generated using CRISPR/Cas9 genome editing [21]. In comparison with Nrf2-WT, the mRNA levels for pirin were 2-fold higher in Nrf2-GoF DLD1 cells, and 50% lower in their Nrf2-knockout counterparts (Figure 1A). This pattern of expression of pirin was similar to that of the classical Nrf2 target protein NAD(P)H:quinone oxidoreductase 1 (NQO1) (Figure 1B), confirming Nrf2 activation. Similar to NQO1, the mRNA levels for pirin were increased 1.5-fold by exposure to TBE-31, and this increase was diminished in Nrf2-knockout DLD1 cells (Figure 1A). The protein levels of pirin were higher in Nrf2-gain-of-function DLD1 cells than in their WT counterparts, and treatment with both TBE-31 and the classical Nrf2 inducer sulforaphane increased the protein levels of pirin in WT cells with no further effect in Nrf2-gain-of-function cells (Figure 1C).

Sulforaphane treatment also increased (by 1.6-fold) the mRNA levels for pirin in the colorectal cancer cell line Caco2 (Figure 1D). Conversely, the mRNA levels for pirin decreased by 90% upon knockout of Nrf2 in the human lung cancer cell line A549, which has constitutively high levels of Nrf2 due to a mutation in Keap1 and hypermethylation of its promoter (Figure 1E). Similarly, the mRNA levels for pirin decreased by 50% upon knockout of Nrf2 in the human osteosarcoma cell line U2OS, in which the levels of Keap1 and Nrf2 are normal (Figure 1F).

In addition to cancer cells, the dependence on Nrf2 for the expression of pirin was also observed in normal human lung fibroblast IMR90 cells, where treatment with TBE-31 or sulforaphane caused similar levels of induction (by ~2.5-fold) of the expression of pirin (Figure 1G). Collectively, these experiments establish the dependence of pirin expression on Nrf2 in human cells.

### 3.2. Pirin Is a Transcriptional Target of Nrf2 in Mice

**Figure 2:**
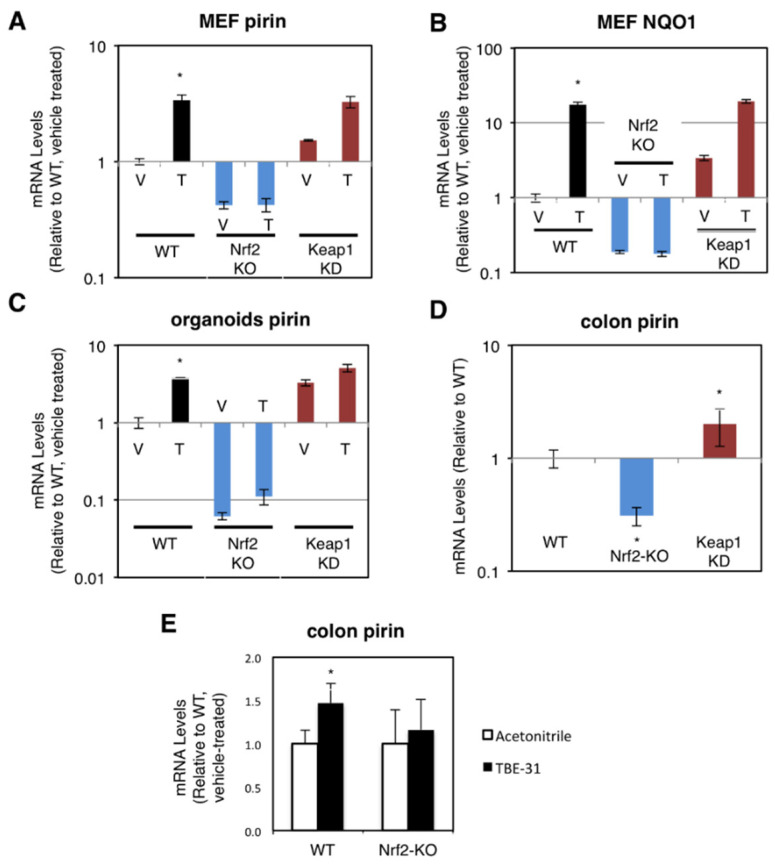
Pirin is a transcriptional target of Nrf2 in mouse cells and in vivo. (**A**,**B**) mRNA levels for pirin (**A**) and NQO1 (**B**) in Nrf2-wild-type (WT), Nrf2-knockout (Nrf2-KO), and Keap1-knockdown (Keap1-KD) mouse embryonic fibroblast (MEF) cells treated with vehicle (V) or 50 nM TBE-31 (T) for 24 h. The TaqMan data were normalized using eukaryotic 18S rRNA (18S) as an internal control. (**C**) mRNA levels for pirin in Nrf2-wild-type (WT), Nrf2-knockout (Nrf2-KO), and Keap1-knockdown (Keap1-KD) mouse intestinal organoid cultures treated with vehicle (V) or 10 nM TBE-31 (T) for 24 h. (**D**) mRNA levels for pirin in the colon of wild-type (WT), Nrf2-knockout (Nrf2-KO), and Keap1-knockdown (Keap1-KD) C57BL6 mice (*n* = 6–8). (**E**) mRNA levels for pirin in the colons of wild-type (WT) and Nrf2-knockout (Nrf2-KO) mice (*n* = 4–5), which had been treated with TBE-31 (5 nmol/g body weight, 3 times, at 24-h intervals, per os, black bars) or vehicle (0.7% DMSO in corn oil, white bars). * *p* < 0.05. The TaqMan data were normalized using mouse ribosomal protein lateral stalk subunit P0 (rplp0) as an internal control.

The dependence on Nrf2 of both basal and inducible expression of pirin and NQO1 was also observed in primary cultures of mouse embryonic fibroblast (MEF) cells, intestinal organoids, and in the murine colon in vivo. For these experiments, we used wild-type (WT), Nrf2-knockout (Nrf2-KO, Nrf2^−/−^) and Keap1-knockdown (Keap1-KD, Keap1^flox/flox^) mice [19,24,27]. Compared to WT, the mRNA levels for pirin were 60% lower and not inducible by TBE-31 in Nrf2-knockout MEF cells, whereas these levels were 1.5-fold higher in their Keap1-knockdown counterparts (Figure 2A). The basal and TBE-31 induced expression of NQO1 in the three genotypes paralleled that of pirin (Figure 2B).

Next, we prepared organoids from the small intestine of WT, Nrf2-KO, and Keap1-KD mice. In agreement with the data in human and MEF cells, compared to WT, the mRNA levels for pirin were 3.3-fold higher in Keap1-knockdown organoids, whereas these levels were >90% lower in their Nrf2-knockout counterparts (Figure 2C). Treatment with TBE-31 induced pirin 3.9-fold in WT organoids, but this induction was greatly diminished in organoids from Nrf2-knockout or Keap1-knockdown mice (Figure 2C).

In the colons of mice from the three genotypes, the expression of pirin was 70% lower and 2-fold higher in Nrf2-knckout and Keap1-knckdown, respectively (Figure 2D). Oral administration of TBE-31 to mice induced the expression of pirin in wild-type animals, but had no effect in their Nrf2-knockout counterparts (Figure 2E). Thus, using genetic and pharmacologic approaches, we have demonstrated that pirin is a transcriptional target of Nrf2 in human colorectal cancer cells, mouse intestinal organoid cultures and in vivo in the murine colon.

### 3.3. Pirin Is Overexpressed in Human Colorectal Tumors

**Figure 3:**
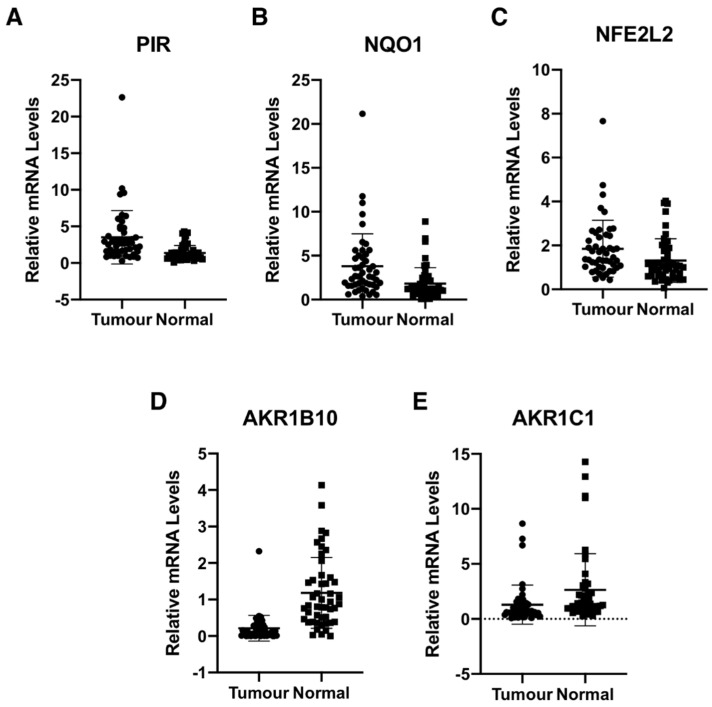
Pirin is overexpressed in human colorectal tumours. mRNA levels for pirin (**A**), NQO1 (**B**), NFE2L2 (**C**), AKR1B10 (**D**), and AKR1C1 (**E**) in matched tumour and normal tissues from human colorectal cancer patients (*n* = 48). *p* < 0.05 in all cases. The TaqMan data were normalized using eukaryotic 18S rRNA (18S) as an internal control.

We determined the gene expression of pirin in 48 primary human colorectal tumours and matched normal tissue from the same patients. Using *18S* as a reference gene and a 2-fold increase or a 50% decrease relative to normal tissue as a cut-off, we found that the mRNA levels for pirin were increased in 30/48 and decreased in 2/48 tumour samples in comparison with their corresponding matched normal tissues (Figure 3A). The mRNA levels for NQO1 were increased in 25/48 tumours and decreased in the same two tumour samples, which also had lower pirin expression (Figure 3B). Based on Spearman and Kendall rank correlation test, the folds change of pirin expression in tumour vs. the corresponding normal tissues correlated with the fold change of NQO1 expression (*p* = 0.001271 and *p* = 0.0008092). Furthermore, when we examined the mRNA levels for Nrf2 in the human tissues, we found them to be increased in 18/48 and decreased in 2/48 tumour samples in comparison with their matched normal tissues (Figure 3C). The correlation test also found that the fold change of pirin expression correlated with the fold change of Nrf2 expression (*p* = 1.49E-05 and *p* = 9.32E-06). Together, these data strongly suggest that the overexpression of pirin in the tumour tissues is dependent on Nrf2.

**Figure 4:**
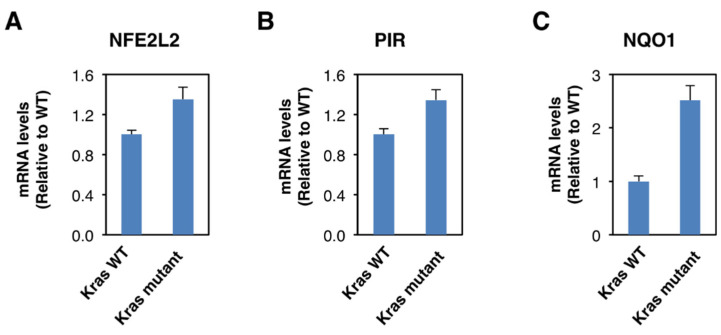
Constitutive activation of K-Ras enhances *NFE2L2* transcription. mRNA levels for NFE2L2 (**A**), pirin (**B**), and NQO1 (**C**) in K-Ras^WT/−^ and K-Ras^G13D/−^ mutant DLD1 cell lines. *p* < 0.05 in all cases. The TaqMan data were normalized using eukaryotic 18S rRNA (18S) as an internal control.

Although Nrf2 is primarily regulated on the protein level, oncogene-induced Nrf2 overexpression has also been reported [28]. Thus, it has been shown that the expression of an endogenous oncogenic allele of K-Ras(G12D) in mouse embryonic fibroblasts increases the transcription of Nrf2 [28]. Therefore, we compared the expression of Nrf2, pirin, and NQO1 in K-Ras^WT/−^ and K-Ras^G13D/−^ DLD1 cell lines. We found that the mRNA levels for Nrf2 were 1.3-fold higher in the oncogenic K-Ras-expressing cell line compared to its wild-type K-Ras-expressing counterpart (Figure 4A), confirming the transcriptional upregulation of Nrf2 under conditions of K-Ras activation. The mRNA levels for pirin (Figure 4B) and NQO1 (Figure 4C) were also increased in the oncogenic K-Ras-expressing cells by 1.3- and 2.5-fold, respectively, in agreement with Nrf2 activation. Together with the analysis of the human tissues, these data indicate that Nrf2 is activated in human colorectal cancers, and this activation is likely mediated by both transcriptional and post-transcriptional mechanisms.

Curiously however, the expression of the aldo-keto reductase family 1 members AKR1B10 and AKR1C1, which are well-established Nrf2 targets in humans, showed the opposite pattern to that of pirin, Nrf2 and NQO1 in the human tissues. Thus, compared to matched normal tissue, the mRNA levels for AKR1B10 and AKR1C1 were decreased in 39/48 and 28/48 tumour samples, respectively (Figure 3D,E). In both cases, the differences between the tumour and normal tissues were statistically significant. This finding suggests that transcriptional mechanism(s) other than regulation by Nrf2 are responsible for the downregulation of the gene expression AKR1B10 and AKR1C1 in human colorectal tumours.

### 3.4. Pirin Does Not Affect the Viability or Migration of DLD1 Colorectal Cancer Cells

**Figure 5:**
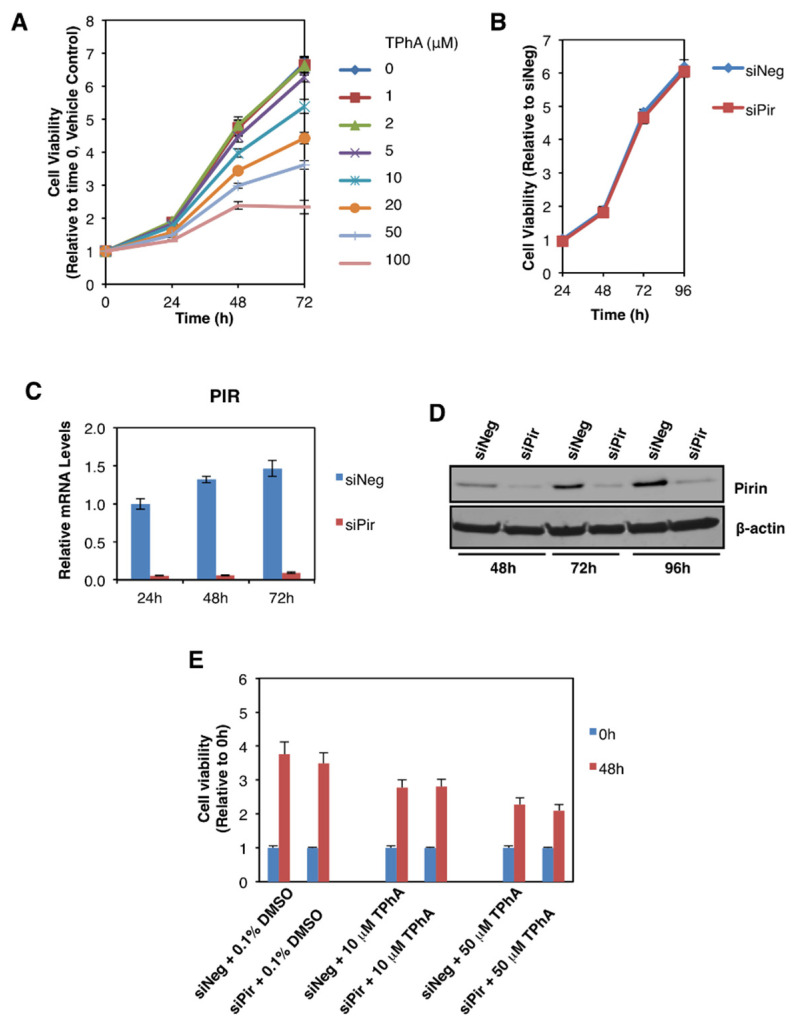
Pirin does not affect the viability of DLD1 colorectal cancer cells. (**A**) DLD1 cells (5 × 10^3^ per well) plated in 96-well plates were treated with the indicated concentration of TphA for 24-, 48- and 72 h. Cell viability was assessed using the Alamar Blue fluorometric assay. (**B**) DLD1 cells (5 × 10^3^ per well) were transfected with 20 nM ON-TARGET plus Smart Pool siRNA against human PIR (siPir) or ON-TARGET plus Non-targeting Control Pool (siNeg). Cell viability was monitored for four days after transfection with Alamar Blue reagent. (**C**) mRNA levels for pirin in DLD1 cells transfected with 20 nM siNeg or siPir for 24-, 48- and 72 h. (**D**) Protein levels for pirin in DLD1 cells transfected with 20 nM siNeg or siPir for 48-, 72- and 96 h. (**E**) DLD1 cells (5 × 10^3^ per well) were transfected with 20 nM siNeg or siPir for 48 h, and then treated with 0.1% DMSO, 10 μM or 50 μM TPhA. Cell viability was assessed using the Alamar Blue fluorometric assay at 0- and 48 h after treatment.

We then addressed the potential functional consequences of the observed pirin upregulation in colorectal tumours. Several reports have implicated pirin as an important factor in cancer cell proliferation, migration, and tumour progression [3,4,5]. Thus, first we asked whether pirin affects the viability of DLD1 colorectal cancer cells using two approaches: (i) pharmacological, by treatment with triphenyl compound A (TPhA), a small molecule inhibitor of pirin [5], and (ii) genetic, by knockdown of pirin using siRNA. Exposure to TPhA concentration-dependently inhibited the viability of DLD1 cells (Figure 5A). By contrast, depleting pirin by siRNA had no effect on cell viability (Figure 5B), in spite of the high efficiency of the knockdown at both the mRNA (Figure 5C) and the protein (Figure 5D) levels. Furthermore, the TPhA-mediated inhibition of cell viability occurred at a similar level in cells transfected with pirin siRNA as it did in siRNA negative control-transfected cells (Figure 5E), strongly suggesting that the inhibitory effect of this compound in our experimental system was pirin-independent.

**Figure 6:**
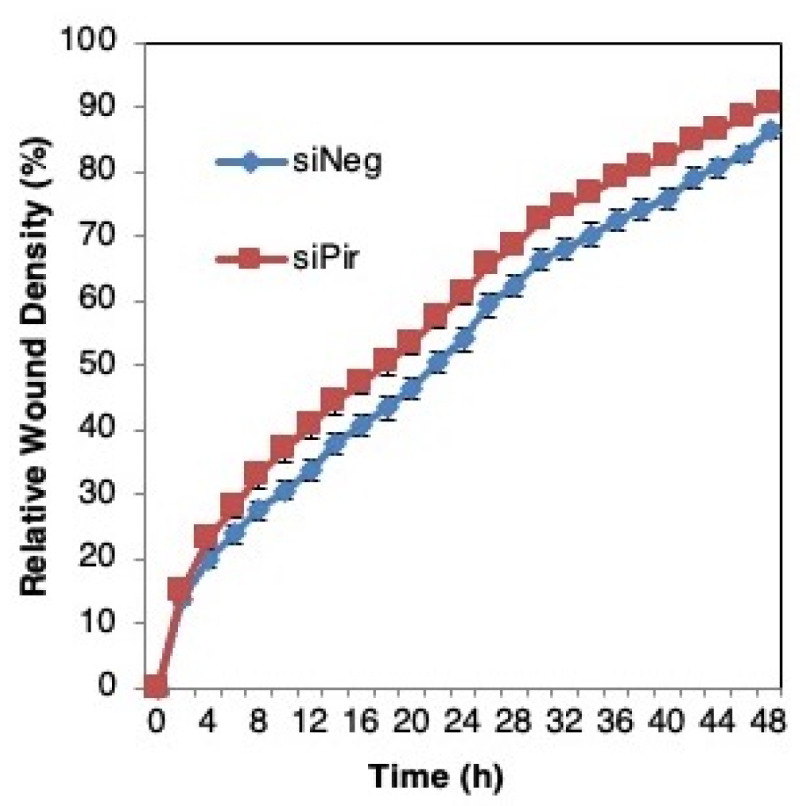
Pirin does not affect the migration of DLD1 colorectal cancer cells. DLD1 cells were transfected with 20 nM ON-TARGET plus Smart Pool siRNA against human PIR (siPir) or ON-TARGET plus Non-targeting Control Pool (siNeg) and grown for 48 h. Transfected cells (1.5 × 10^4^ cells per well, 6 replicate wells for each condition) were seeded in 96-well ImageLock plates (Essen BioScience) and grown for a further 48 h to confluence. Cell migration was monitored at 2-h intervals for two days using IncuCyte™.

We then asked whether pirin affects the migration of DLD1 colorectal cancer cells using a well-established functional wound-healing assay. To this end, cells were transfected with pirin siRNA or siRNA negative control as above, grown to confluency, and subsequently serum starved to prevent cell proliferation. A uniform scratch wound was then introduced in the confluent cell monolayer in a tightly-controlled manner, and the resulting wound closure was monitored by real-time imaging. Depleting pirin by siRNA had no significant effect on wound closure (Figure 6), suggesting that under these experimental conditions, pirin does not affect cell migration.

## 4. Discussion

A number of studies have documented the tumour-promoting roles of pirin in human cervical, lung, oral, and skin cancers, and have been recently reviewed [29]. However, the status of pirin in colorectal cancer has not been documented. Our study extends the current knowledge by showing that pirin is overexpressed in human colorectal cancer in comparison with matched control tissue. Our functional studies show that pirin depletion does not affect the viability or migration of DLD1 colorectal cancer cells, and thus the functional significance of pirin overexpression in colorectal cancer requires further investigation. In this context, it is notable that a recent bioinformatic analysis has shown that pirin is functionally associated with six proteins, including Nck-associated protein 1 (NCKAP1) and cytoplasmic protein (NCK1) [29]. Both NCKAP1 and NCK1 have been implicated in facilitating cell invasion in breast cancer [30,31], suggesting that one of the functions of pirin overexpression is to promote cancer cell invasion, and that the invasion-promoting activity of pirin could be mediated through its functional interactions with NCKAP1 and/or NCK1.

These findings suggest that inhibition of pirin could be beneficial for cancer treatment and justify the ongoing search for pirin inhibitors. One of the first discovered compounds, which binds to pirin, was triphenyl compound A (TPhA) [5]. These authors showed that TPhA inhibits melanoma cell migration. Subsequently, pirin was identified as a high affinity molecular target of bisamide (CCT251236) and shown to decrease tumour growth in a human ovarian carcinoma xenograft model [32]. Most recently, pirin was also identified as a molecular target of a series of compounds, which have been shown to inhibit melanoma metastasis and bleomycin-induced skin fibrosis [33]. However, although all of these compounds bind pirin, their specificity for pirin inhibition is unknown. In our study TPhA decreased the viability of pirin-proficient and pirin-deficient colorectal cancer cells equally well, strongly suggesting that this compound has additional targets and supporting the need for the identification and development of more specific pirin inhibitors.

The overexpression of pirin in human colorectal cancer correlated with the overexpression of Nrf2 and the classical Nrf2 target gene, NQO1, suggesting that increased transcription of both PIR and NQO1 is a consequence of Nrf2 activation. This is in agreement with our previous report that the expression of a number of Nrf2 transcriptional targets was increased in tumour tissues of colorectal cancer patients, and that high levels of nuclear Nrf2 correlate with a poor patient prognosis [34]. Notably however, the current study revealed that compared to matched normal tissue, the mRNA levels for AKR1B10 and AKR1C1 were decreased. This finding was unexpected, because the genes encoding the human aldo-keto reductases (AKRs) are considered to be some of the most overexpressed genes in response to Nrf2 activation [35]. Moreover, AKR1B10 and AKR1C1 are overexpressed in non-small cell lung cancer and strongly correlate with Nrf2 activation [36].

Although initially surprising, the reduced expression of AKR1B10 in the tumours is consistent with the previously reported decreased AKR1B10 expression in human colorectal cancer [37,38] and the decrease in the mRNA levels for AKR1B10 in sera of colon cancer patients in comparison with sera of healthy donors [39]. In addition to Nrf2, AKR1B10 is transcriptionally regulated by p53 [37,40], in agreement with the upregulation of the AKR1B10 mRNA by chemotherapeutic agents in normal diploid cells and in cancer cells expressing wild type p53, and its downregulation in cancer cells harbouring mutant p53 [39]. Because p53 is commonly mutated in colorectal cancer [41], the observed downregulation of AKR1B10 in our study is most likely due to loss of the normal function of p53. Considering that the AKRs have key roles in the detoxification of aldehydes, such as the lipid peroxidation product 4-hydroxy-2-nonenal, as well as nicotine-derived nitrosaminoketones [35], the decrease in the AKR1B10 levels is expected to increase the toxicity and carcinogenicity of these damaging agents. Additionally, it has been shown that loss of AKR1B10 promotes the proliferation and migration of colorectal cancer cells via activation of fibroblast growth factor 1 (FGF1) [38]. Thus, the downregulation of AKR1B10 in the colon may be a contributing factor to the development of colorectal cancer.

The molecular cloning of pirin was reported in 1997 by Wendler et al. [42]. A decade later, Gelbman et al. [43] found a 2-fold increase in pirin expression in the airway epithelium of smokers compared to non-smokers and Hübner et al. [8] identified pirin as a “smoking-responsive Nrf2-modulated gene”. Brzoska et al. [9] demonstrated that the transcriptional regulation of pirin by Nrf2 in HeLa cells is largely dependent on a highly conserved ARE located 281 bp downstream of the transcription start site, and the binding of Nrf2 to this ARE. Our findings further support the role of Nrf2 in the regulation of pirin expression by showing that genetic or pharmacologic Nrf2 activation enhances pirin expression, whereas Nrf2 deletion or disruption has the opposite effect in several experimental systems, and that the overexpression of pirin in human colorectal tumours correlates with Nrf2 activation.

An interesting corollary of our study is the unexpected downregulation of AKR1B10 and AKR1C1 expression in human colorectal tumours. Increased expression levels of both AKR1B10 and AKR1C1 are widely accepted as markers of Nrf2 activation in humans, and our findings suggest that, in addition to Nrf2, the regulation AKR1B10 (and possibly also AKR1C1) by p53 should be considered. Keeping in mind the critical need for biomarkers of Nrf2 activation in the drug development of pharmacological Nrf2 activators, our finding highlights the critical importance of rigorous validation and careful consideration of the specific biological context in the design of clinical trials.

## 5. Conclusions

Pirin, an evolutionary conserved non-heme Fe-containing family member of the cupin superfamily of proteins, is regulated by Nrf2 in human and mouse cells and in the mouse colon in vivo. Moreover, pirin is overexpressed in human colorectal tumours where pirin expression correlates with Nrf2 activation, suggesting Nrf2 dependence. The depletion of pirin in the human colorectal cancer cell line DLD1 does not affect cell viability or migration. Understanding the functional consequences of the observed pirin upregulation in colorectal cancer requires further investigation.

## Figures and Tables

**Table 1:** Patient demographics and tumour pathology.

		Female	Male	Total
Number of patients		21	27	48
Age (median (range))		79 (52–91)	68 (54–82)	73.5 (52–91)
Dukes’s stage	A	0	7 (14.6%)	7 (14.6%)
	B	14 (29.2%)	4 (8.3%)	18 (37.5%)
	C	7 (14.6%)	16 (33.3%)	23 (47.9%)
	D	0	0	0
Differentiation	Moderate	17 (35.4%)	22 (45.8%)	39 (81.25%)
	Poor	4 (8.3%)	5 (10.4%)	9 (18.75%)

**Table 2:** Gene Expression Assay IDs.

Gene Name	Assay ID
Pir (mouse)	Mm01721878_m1
Nqo1 (mouse)	Mm01253561_m1
Rplp0 (mouse)	Mm00725448_s1
PIR (human)	Hs01125822_m1
NQO1 (human)	Hs00168547_m1
AKR1B10 (human)	Hs00252524_m1
AKR1C1 (human)	Hs04230636_sH
18S (human)	Hs99999901_s1
HPRT1 (human)	Hs02800695_m1

## Data Availability

All data are contained within the article.
